# A prognostic model based on necroptosis-related genes for prognosis and therapy in bladder cancer

**DOI:** 10.1186/s12894-023-01175-z

**Published:** 2023-01-28

**Authors:** Zeyi Wang, Zhengnan Huang, Xiangqian Cao, Fang Zhang, Jinming Cai, Pengfei Tang, Chenkai Yang, Shengzhou Li, Dong Yu, Yilin Yan, Bing Shen

**Affiliations:** 1 Department of Urology, Shanghai General Hospital of Nanjing Medical University, Shanghai, 200080 China; 2 Department of Urology, Tongji Hospital, School of Medicine, Tongji University, Shanghai, 200065 China; 3 Department of Urology, Shanghai General Hospital, Shanghai Jiaotong University School of Medicine, Shanghai, 200080 China; 4 Department of Precision Medicine, Center of Translation Medicine, Naval Medical University, Shanghai, 200082 China; 5 Department of Urology, Shanghai General Hospital, Shanghai Jiaotong University School of Medicine, Shanghai, 200080 China

**Keywords:** Bladder cancer, Necroptosis, Tumor immune microenvironment, Prognostic model, Immunotherapy, Chemotherapy

## Abstract

Bladder cancer, one of the most prevalent malignant cancers, has high rate of recurrence and metastasis. Owing to genomic instability and high-level heterogeneity of bladder cancer, chemotherapy and immunotherapy drugs sensitivity and lack of prognostic markers, the prognosis of bladder cancer is unclear. Necroptosis is a programmed modality of necrotic cell death in a caspase-independent form. Despite the fact that necroptosis plays a critical role in tumor growth, cancer metastasis, and cancer patient prognosis, necroptosis-related gene sets have rarely been studied in bladder cancer. As a result, the development of new necroptosis-related prognostic indicators for bladder cancer patients is critical. Herein, we assessed the necroptosis landscape of bladder cancer patients from The Cancer Genome Atlas database and classified them into two unique necroptosis-related patterns, using the consensus clustering. Then, using five prognosis-related genes, we constructed a prognostic model (risk score), which contained 5 genes (ANXA1, DOK7, FKBP10, MAP1B and SPOCD1). And a nomogram model was also developed to offer the clinic with a more useful prognostic indicator. We found that risk score was significantly associated with clinicopathological characteristics, TIME, and tumor mutation burden in patients with bladder cancer. Moreover, risk score was a valid guide for immunotherapy, chemotherapy, and targeted drugs. In our study, DOK7 was chosen to further verify our prognosis model, and functional assays indicated that knockdown the expression of DOK7 could prompt bladder cancer proliferation and migration. Our work demonstrated the potential role of prognostic model based on necroptosis genes in the prognosis, immune landscape and response efficacy of immunotherapy of bladder cancer.

## Background

Bladder cancer (BLCA), one of the most prevalent malignant cancers, has high rate of recurrence and metastasis, and there is a strong male predominance (male to female ratio 3:1) [1]. It is estimated that male BLCA new cases and deaths will rank fourth and eighth in the United States, respectively [1]. During 1975 to 2018, there is no significant change in the incidence of BLCA in men, but the incidence of other common tumors such as lung cancer decreased year by year [1]. BLCA present as muscle-invasive bladder cancer (MIBC), non-muscle-invasive bladder cancer (NMIBC) and metastatic diseases, approximately 75% of newly diagnosed BLCA patients present with NMIBC [2]. Radical cystectomy and cisplatin-based neoadjuvant chemotherapy are commonly options to treat BLCA especially MIBC. And new immunotherapy approaches are improving outcomes [3]. Yet, despite great progress in diagnosis and treatment, BLCA still shows a poor prognosis in high rates of metastasis and recurrence. The reason may be genomic instability and high-level heterogeneity of BLCA, chemotherapy and immunotherapy drugs sensitivity and lack of prognostic markers [4–6]. Therefore, there is an essential need to develop potential strategy to improve efficacy.

Necroptosis is a programmed modality of necrotic cell death in a caspase-independent form [7]. It has been reported that necroptosis is mainly mediated by receptor-interacting protein kinase 1 (RIPK1), RIPK3 and mixed lineage kinase domain-like pseudo kinase (MLKL), while necrostatin-1 (Nec-1) inhibits it [8]. Different with apoptosis, morphological features of necroptosis are typified as cell membrane rupture, organelle swelling and increasingly translucent cytoplasm [9, 10]. With the rupture of plasma membrane, cell contents are released which could lead to the exposure of damage-associated molecular patterns (DAMPs) and powerful inflammatory responses [11]. The dual-effects of necroptosis on cancer have been proven [12]. On the one hand, with the downregulation of necroptotic factors, it could cause a worse prognosis [13–15]. On the other hand, the upregulation of necroptotic factors also could lead to a worse prognosis and promote oncogenesis [16, 17]. Chemotherapy failure is a hard but common problem, and drug resistance induced by apoptosis plays a major role in it [12, 18]. There are lot of reports that chemotherapeutic agents could trigger necroptosis in cancer [19, 20], so we believe induced necroptosis may provide an effective therapy strategy in anti-cancer. Furthermore, RIPK1 expression and NF-κB activation during necroptosis could play a role of antitumor immunity by activate CD8^+^T cells [21, 22]. Thus, necroptosis may also be of vital potential in the prognosis and therapy of BLCA.

Herein, we used multi-omics analysis to analysis the differences between necroptosis-related patterns based on the expression patterns of necroptosis-related genes (NRGs). Next, to predict the OS of BLCA patients, a prognostic model was constructed and validated its prognostic accuracy. In addition, we also built a nomogram model to provide clinical BLCA patients with precise and stable prognostic forecasts. Mutation profile, immune cell infiltration and immunotherapeutic and chemotherapeutic efficacy were also explored. Our study may provide a prognosis predicter and novel therapeutic targets for BLCA patients.

## Methods

### Obtaining and processing data

The datasets we used were all publicly available. Data of clinical information, somatic mutation and gene expression were obtained from The Cancer Genome Atlas (TCGA) data portal (https://portal.gdc.cancer.gov/). Data of clinical information and gene expression from the GEO database (https://www.ncbi.nlm.nih.gov/geo/, GSE13507) were used as an external test set. Next, in order to normalize the raw expression data, we used Robust Multiarray Average [23]. We obtained 74 NRGs from previous study [24]. Immunohistochemical (IHC) staining of DOK7 between BLCA and normal tissues were directly visualized by HPA (https://www.proteinatlas.org) [25].

### Consensus clustering

In our study, we used consensus clustering to classify TCGA-BLCA cohort based on the expression of prognosis-related NRGs mRNA [26]. According to methods of previous study [27], the ideal cluster number was found to be k = 2. The classification was verified by PCA based on the expression of prognosis-related NRGs mRNA of TCGA-BLCA cohort.

### Tumor immune microenvironment evaluation

ssGSEA, CIBERSORT, and ESTIMATE were used in R to evaluate the TIME status of each BLCA sample. The enrichment scores of immune functions and immune cells were quantified using ssGSEA. ESTIMATE was applied for assess of the stromal, ESTIMATE and immune score. 22 tumor-infiltrating immune cells (TIICs) were valued by CIBERSORT algorithm.

### Functional enrichment analysis of DEGs between necroptosis-related patterns

After consensus clustering, we examined the distinction of biological pathways of different expression gene set (DEGs) between necroptosis-related patterns through KEGG [28] and GO and pathway enrichment analyses. The GO terms were in the cellular component (CC), biological process (BP) and molecular function categories (MF).

### Establishment and validation of the prognostic model

We obtained the coefficient and selected the minimum criteria threshold for further screening 7 genes using LASSO Cox regression analysis. Eventually, the stepwise regression analysis provided a more practical and optimal model with five genes. In addition, the risk score formula was as follows:

$$Risk\;score = \mathop \sum \limits_{n = 1}^{i} \left( {exp\;Genei \times coefficient\;Genei} \right)$$

Based on the formula, we chose the mean value of risk score to divide TCGA-BLCA cohort into high- and low-risk groups. Next, the prognostic model's prediction ability was evaluated using the receiver ROC curve and Kaplan–Meier analysis. The GSE13507 datasets used the same approaches to validate the model.

### Calculation of tumor microenvironment cell infiltration

XCELL, MCPCOUNTER, CIBERSORT, TIMER, EPIC, QUANTISEQ, and CIBERSORT-ABS were utilized to assess the relative proportions of infiltrating immune cells [29]. Spearman’s rank correlation analysis was used when groping the correlation between the immune infiltrated cells and the risk score.

### Evaluation of chemotherapy and immunotherapy drugs' efficacy

The pRRophetic software package was used to calculate the half-maximal inhibitory concentration (IC50) values of commonly used chemotherapy and immunotherapy drugs.

### Cell culture and transfection

The human bladder cancer cell line 5637 was bought from the Cell Bank of Type Culture Collection of Chinese Academy of Sciences (Shanghai, China) and cultured in RPMI1640 supplemented with 10% FBS and penicillin–streptomycin (100 U/mL) in a humidified atmosphere of 5% CO2 at 37 °C. For the knockdown assay, small interfering RNA targeting DOK7 (siDOK7) was applied, and scramble siRNAs (siNC) as the negative control. The siRNA sequences targeting DOK7 was as follows: DOK7 siRNA sequence 5′-CUGGUCUACAAGGACAAGUTT-3′; siNC (noncoding control): 5′-UUCUCCGAACGUGUCACG U-3′.

### Cell counting assay-8(CCK-8), wound healing assay and transwell migration assay

CCK8 assay (MCE, American) was used to analyze cell proliferation. 1 × 10^3^ cells were cultured in 96-well plates for 24 h, 48 h and 72 h. Then, after incubation, 10 μL of CCK8 reagent was added into every well of 96-well plates and incubated for 2 h. After incubation of CCK8 reagent, we measured the OD value at 450 nm.

For the wound healing assay, the cells were plated in six-well plates, and cultured until 90% confluent. The confluent monolayer was wounded with a 200-μL pipette tip, and the unattached cells were removed. The scratches were observed at 0 h and 24 h after incubation of the monolayers in the FBS-free medium.

For migration assay, 1 × 10^4^ bladder cancer cells were seeded on the upper 24-well transwell chambers (Corning) and culture for 24 h. After incubation, the cells move to the bottom of the 24-well chamber, followed by fixing with 4% formaldehyde and dyeing with crystal violet reagent.

### Statistical analysis

All statistical analyses were performed using the R software (version 4.1.2). The Wilcoxon rank-sum test, paired samples *t*-test, and Kruskal–Wallis test were employed to validate the statistical difference between two groups or more than two groups, respectively. The correlation coefficients between tumor mutation burden (TMB), immune checkpoint genes (ICGs) expression, and risk score were calculated by Spearman’s correlation analysis. *P* value < 0.05 was defined as a statistically significant standard.

## Results

### Consensus clustering of necroptosis-related patterns in TCGA-BLCA cohort

**Fig. 1:**
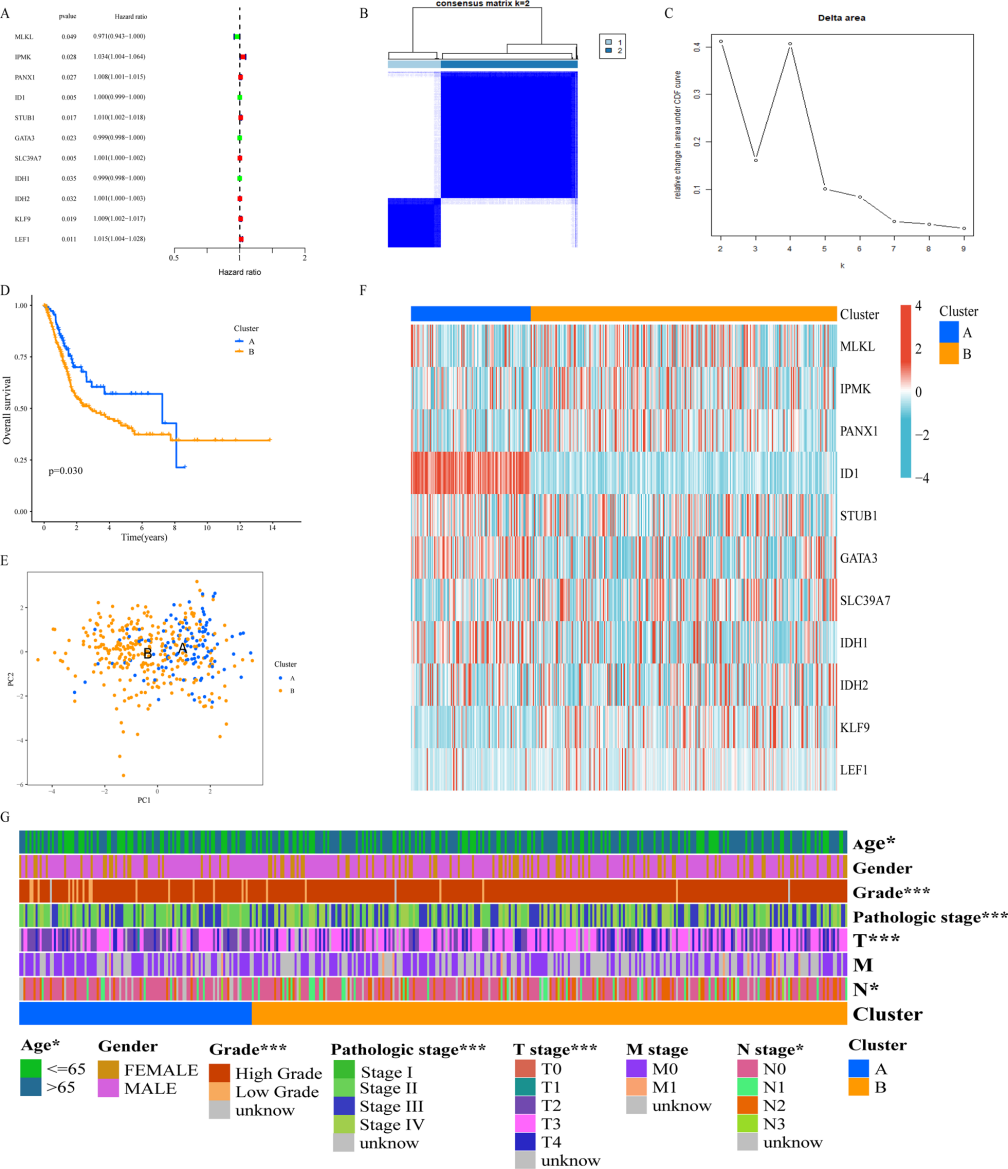
Consensus Clustering of Necroptosis-Related Patterns in TCGA-BLCA cohort. **A** Prognosis-related NRGs selected by univariate COX regression analysis. **B** Consensus matrix heatmap defining two clusters (k = 2) and their correlation area. **C** Cumulative distribution function curve. Kaplan–Meier analysis (**D**) and PCA analysis (**E**) of necroptosis-related patterns. **F** NRGs with a prognosis-related expression profile. **G** Clinical relevance of necroptosis-related patterns. *p < 0.05; **p < 0.01; ^***^p < 0.001

Using univariate COX regression, we selected 11 prognosis-related NRGs from 74 NRGs (Fig. 1A). Based on 11 prognosis-related NRGs, we performed consensus clustering on TCGA-BLCA cohort. According to cophenetic coefficients, k = 2 was found to be the ideal cluster (Fig. 1B, C). Ultimately, we identify two different necroptosis-related patterns named cluster A and cluster B. After clustering, Kaplan–Meier analysis demonstrated that cluster A has significantly better survival time than cluster B (Fig. 1D). Next, we performed PCA to show the distinction between cluster A and cluster B at the 11 prognosis-related NRGs transcription level (Fig. 1E). The transcription profile of 11 prognosis-related NRGs was presented as heatmap (Fig. 1F). As Fig. 1G showed that the distribution of age, grade, pathologic stage, T stage and N stage were significantly distinct between cluster A and cluster B by chi-square test.

### Tumor immune microenvironment of necroptosis-related patterns

**Fig. 2:**
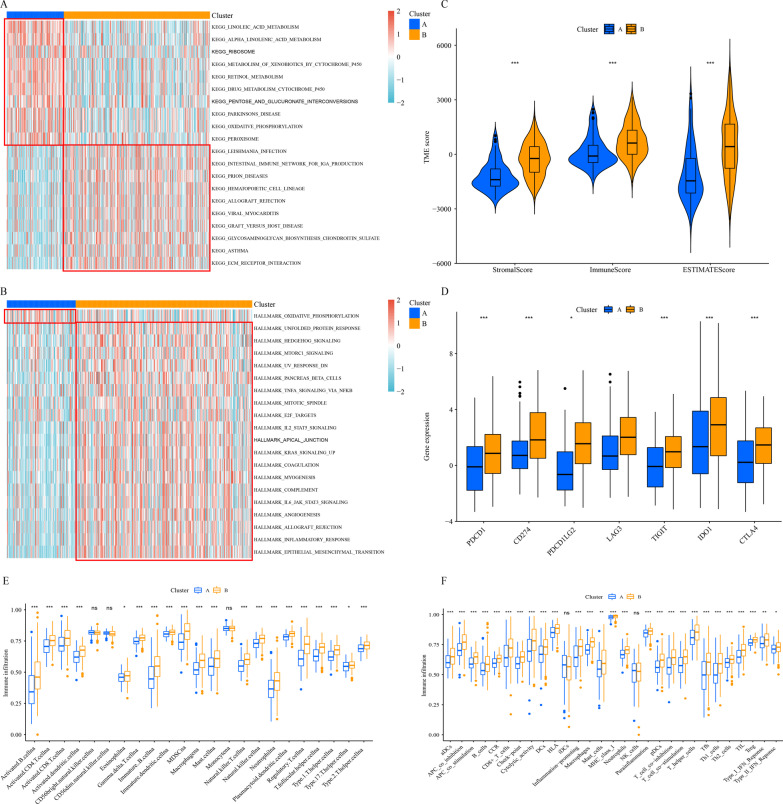
Correlation between the tumor immune microenvironment and necroptosis-related patterns. Heatmap of GSEA analysis results based on KEGG gene set (**A**) and HALLMARK gene set (**B**). **C** A comparison of stromal, immune, and ESTIMATE scores. **D** Differential analysis of ICGs expression. **E** Infiltration of 23 TIICs in two necroptosis-related patterns. **F** Enrichment scores of immune-related functions in two necroptosis-related patterns. *p < 0.05; **p < 0.01; ***p < 0.001, ns = no significance

By using GSEA analysis, we confirmed that cluster A has higher concentration in metabolism, while cluster B has a higher concentration in carcinogenic-related pathways and immune-related diseases (Fig. 2A, B). To distinguish the associations between tumor immune microenvironment and two subtypes, we first using ESTIMATE to calculated the tumor microenvironment composition (Fig. 2C). The StromalScore, ImmuneScore, and ESTIMATEScore of cluster A were all notably lower than those in cluster B. In addition, several immune checkpoints expression, including PDCD1, CD274, PDCD1LG2, IDO1, LAG3, TIGIT and CTLA4, were higher in cluster B (Fig. 2D). Moreover, the infiltration of B cells, eosinophilna, T cells, macrophages, dendritic cells and neutrophils in cluster B were notably higher than those in cluster A (Fig. 2E). Uniformly, almost all immune functions were highly expressed in cluster B (Fig. 2F).

**Fig. 3:**
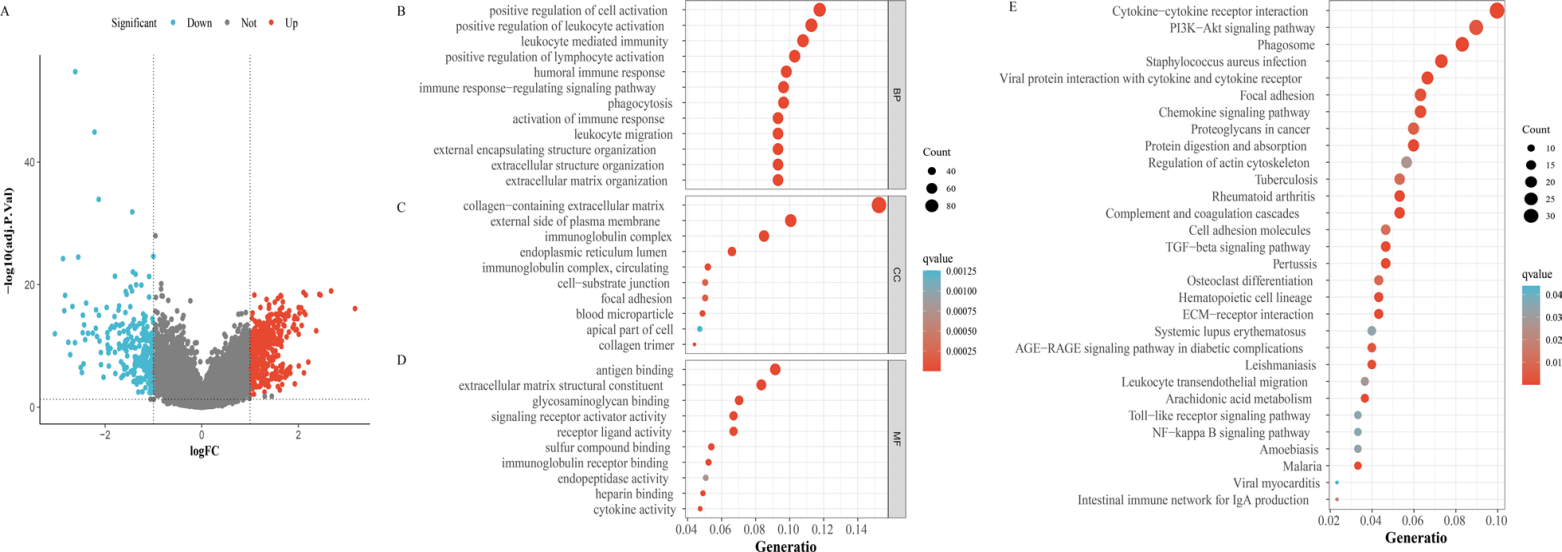
Functional enrichment analyses of DEGs between necroptosis-related patterns. **A** DEGs between necroptosis-related patterns**.** Biological process (**B**), cellular component (**C**), molecular function (**D**), and KEGG pathways (**E**)

Figure 3A showed the DEGs between necroptosis-related patterns by volcano plot. Functional enrichment analyses of DEGs between two subtypes were applicable to grope diversities the molecular diversity. GO analysis indicated that DEGs were mainly involved in positive regulation of cell activation and multiple immune-related biological processes (Fig. 3B). Cellular Components were mostly located in collagen − containing extracellular matrix, external side of plasma membrane and immunoglobulin complex (Fig. 3C). Molecular functions were mostly concentrated in antigen binding, extracellular matrix structural constituent and glycosaminoglycan binding (Fig. 3D). Consistently, the DEGs were related to several immune-related pathways, such as the chemokine signaling pathway, TGF-β signaling pathway and NF-κB signaling pathway (Fig. 3E).

### Establishment and validation of the prognostic model in TCGA-BLCA cohort

**Fig. 4:**
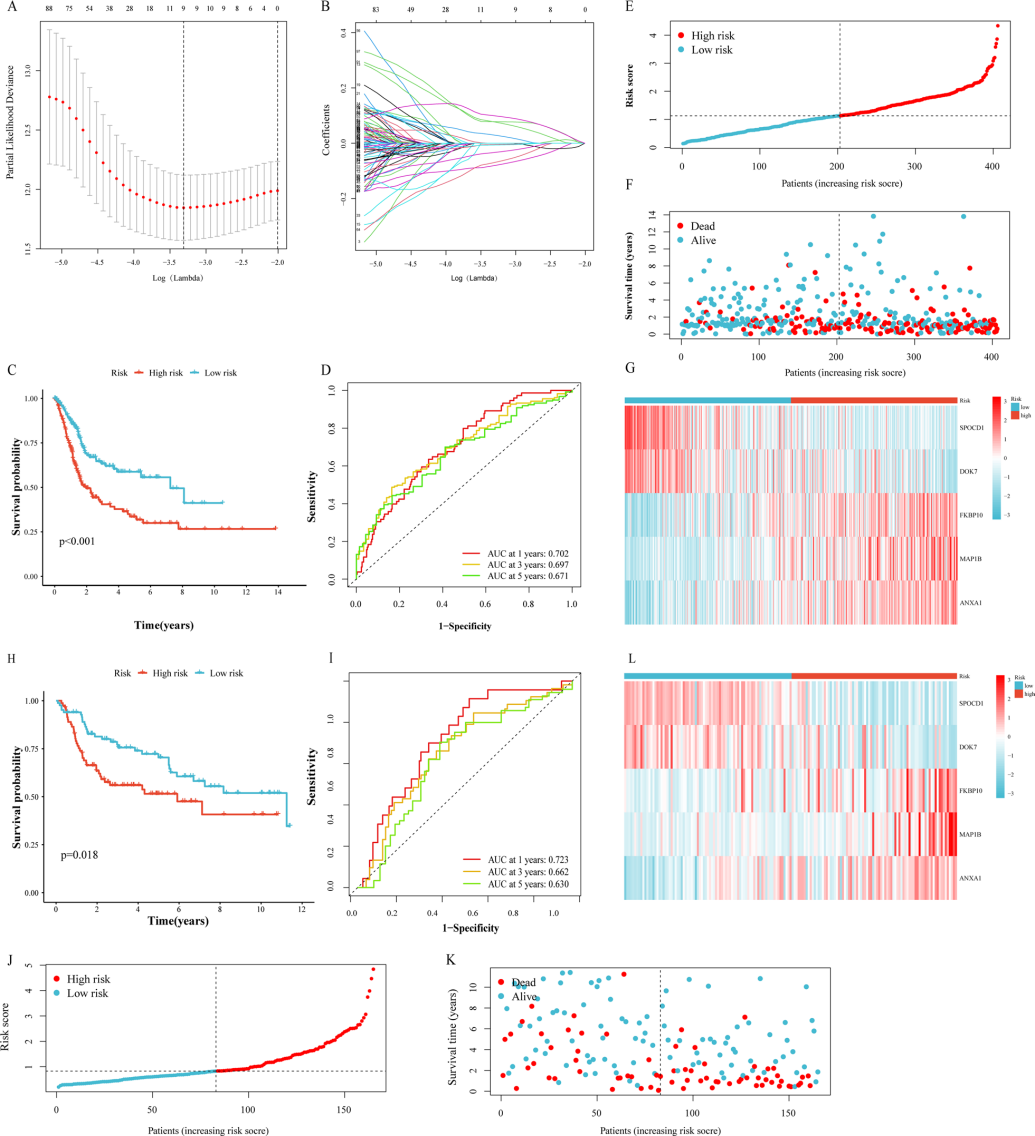
Construction and validation of the prognostic model based on the TCGA-BLCA cohort and GSE13507. **A**, **B** LASSO COX regression analysis. Kaplan–Meier analysis (**C**), time-dependent ROC curve (**D**), risk score distribution (**E**), heatmap of survival statue (**F**), and heatmap of NRG expression profile (**G**) based on the TCGA-BLCA cohort. Kaplan–Meier analysis (**H**), time-dependent ROC curve (**I**), risk score distribution (**J**), heatmap of survival statue (**K**), and heatmap of NRG expression profile (**L**) based on the GSE13507

Based on the necroptosis-related patterns and prognosis-related genes between two subtypes, we established a prognostic model to obtain a metrics that could correctly predict the clinical survival of BLCA patients. The minimum threshold for further screening 9 genes was determined using LASSO Cox regression analysis (Fig. 4A, B). After optimizing the model with stepwise regression analysis, only five genes remain: ANXA1, DOK7, FKBP10, MAP1B and SPOCD1. We also obtained a quantitative metrics: Risk score = (FKBP10 expression × 0.13145) + (MAP1B expression × 0.13152) + (ANXA1 expression × 0.16761) − (SPOCD1 expression × 0.14438) − (DOK7 expression × 0.14033). Based on the formula and the mean value of risk score, we divided patients into the high- and low-risk group. According to Kaplan–Meier analysis, the OS of BLCA patients in the low-risk group was significantly better than that of the high-risk group (Fig. 4C). Additionally, the AUCs for OS survival at 1, 3 and 5 years were 0.702, 0.697 and 0.671, respectively (Fig. 4D). Figure 4E, F showed that the proportion of deaths were positively associated with risk scores. The expression of SPOCD1 and DOK7 were negatively correlated with risk score, whereas FKBP10, MAP1B and ANXA1 were increased with the increasing of risk score (Fig. 4G).

Besides, we used GSE13507 as a test set to further validate the accuracy of risk score in BLCA patients. We used the same formula to quantify the samples in the test set and group them with the same cutoff values. Kaplan–Meier analysis indicated high risk score significantly correlated with poor OS (Fig. 4H). The AUCs for OS survival at 1, 3 and 5 years were 0. 723, 0. 662 and 0. 630, respectively (Fig. 4I). Risk score distribution, survival status and expression profile heatmaps showed similar tendencies to the training set (Fig. 4J–L). Hence, we believe that the prognostic model could be an accurate and effective risk factor for BLCA.

### Clinical relevance of the prognostic model

**Fig. 5:**
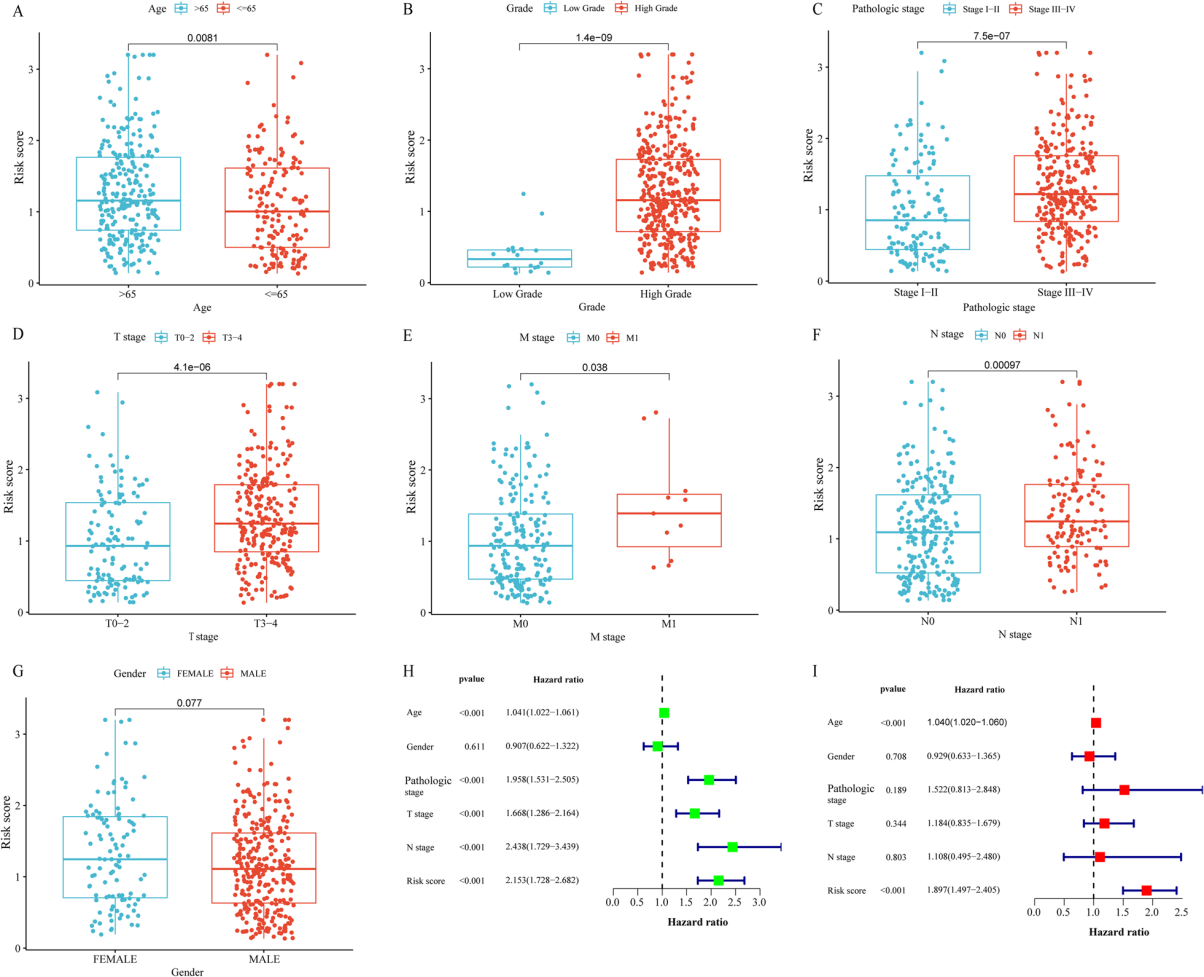
Clinical relevance of the Prognostic model. **A–G** Risk score differences between risk score defined groups of clinicopathological parameters, including age (**A**), grade (**B**), pathologic stage (**C**), T stage (**D**), M stage (**E**), N stage (**F**) and gender (**G**). Univariate (**H**) and multivariate (**I**) Cox regression analysis of risk score and clinicopathological parameters. *p < 0.05; **p < 0.01; ***p < 0.001, ns = no significance

We assessed the connection between clinicopathological parameters and risk score to further investigate the clinical relevance of the prognostic model. Just as Fig. 5A showed that patients older than 65 years scored higher than patients less than or equal to 65 years. And with the grade, pathologic stage, and TNM stages progressed, the risk score increased significantly (Fig. 5B–F). But there was no statistical distinction between the gender groups (Fig. 5G).

Furthermore, we used univariate and multivariate Cox regression analysis to see if risk score was a reliable prognostic factor for BLCA patients. In univariate Cox regression analysis, age, pathological stage, T stage, N stage, and risk score were identified as risk factors (Fig. 5H). The risk score was subsequently validated using multivariate Cox regression analysis, indicating that it may be utilized as a reliable independent predictive indicator for BLCA patients (Fig. 5I).

### Construction of a nomogram model based on the prognostic model

**Fig. 6:**
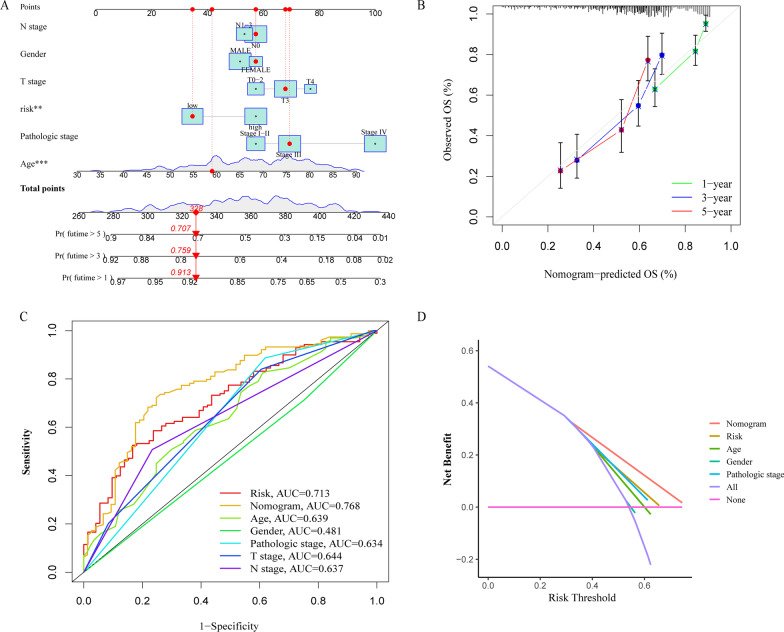
Nomogram model construction. **A** Nomogram predicting the likelihood of OS in 1, 3, and 5 years. **B** Calibration curves for assessing the suitability of the nomogram model in 1, 3 and 5 years. **C** ROC curves of the nomogram, risk and its constituent variables (age, gender, pathologic stage, T stage, N stage) for predicting OS. **D** DCA curves

Based on the above results, patients of high- and low-risk group were assigned the corresponding scores, and the other predicters (N stage, gender, T stage, pathologic stage and age) were also assigned scores respectively. Based on that, we constructed a nomogram model (Fig. 6A). It was show that the 59 years old female patient we select belongs to the low-risk group, at T3N0 stages and pathologic stage III. The assigned scores of the patient were 35, 42, 57, 57, 68 and 69, respectively, with final overall scores of 328. Survival rates were 0.913, 0.759, and 0.707 after 1-, 3-, 5-year, respectively. Next, the calibration plots also confirmed the excellent predictive accuracy of the nomogram model (Fig. 6B).

Next, Fig. 6C presented the ROC curves of the nomogram and its constituent variables for OS. The AUCs of the nomogram and risk for predicting OS survival rate were 0.768 and 0.713, respectively. Consistently, DCA curves demonstrated that risk and nomograms have higher clinical application than pathological staging, age and gender in predicting patient OS (Fig. 6D).

### Correlation between tumor immune microenvironment and the prognostic model

**Fig. 7:**
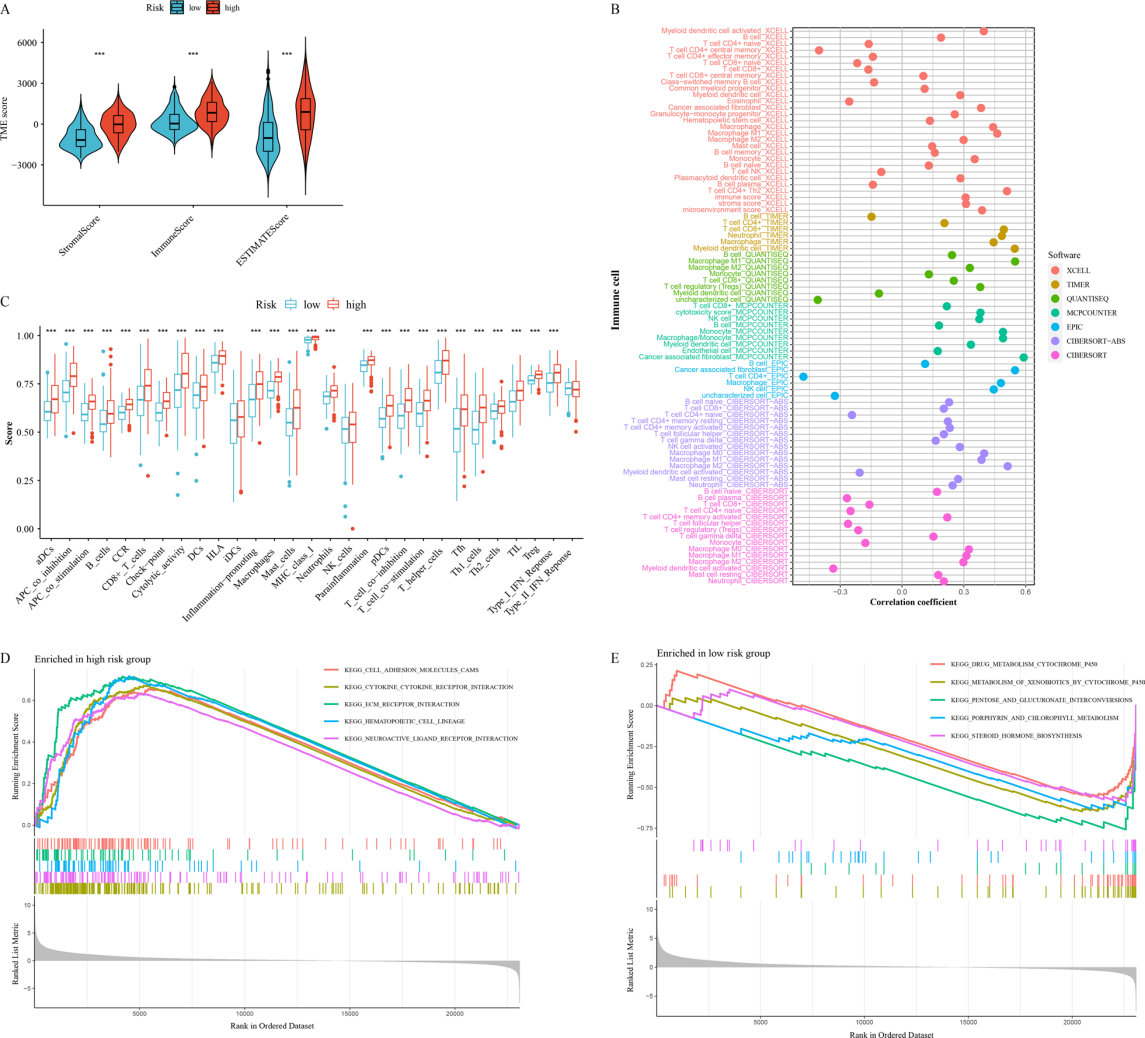
Correlation between the prognostic model and tumor immune microenvironment. **A** A comparison of stromal, immune, and ESTIMATE scores. **B** The relationship between immune cells and risk Score. Each color represented a distinct algorithm. **C** Enrichment scores for immune-related functions in groups defined by risk scores. GSEA analyses based on KEGG in the high-risk group (**D**) and the low-risk group (**E**). *p < 0.05; **p < 0.01; ***p < 0.001, ns = no significance

We further analyzed the correlation between TIME and prognostic model. Using the ESTIMATE algorithm, we first calculated the difference in TME score between high- and low-risk groups (Fig. 7A). The Wilcoxon rank-sum test advised that StromalScore, ImmuneScore and ESTIMATEScore in the low-risk group are significantly lower compare to the high-risk group. The correlation between immune cells and risk score was calculated as well (Fig. 7B). ssGSEA suggested that the majority of immune-related functions were strongly concentrated in the high-risk group (Fig. 7C). Then, GSEA was utilized to investigate potential biological processes and signal pathways. Based on the KEGG gene set, we noticed the concentration level of high-risk group in cell adhesion molecules cams, ECM receptor interaction, cytokine-cytokine receptor interaction, hematopoietic cell lineage and neuroactive ligand receptor interaction (Fig. 7D). While the concentration level of low-risk group in drug metabolism-cytochrome P450, metabolism of xenobiotics by cytochrome p450, Pentose and glucuronate interconversions, Porphyrin and chlorophyll metabolism and steroid hormone biosynthesis (Fig. 7E).

**Fig. 8:**
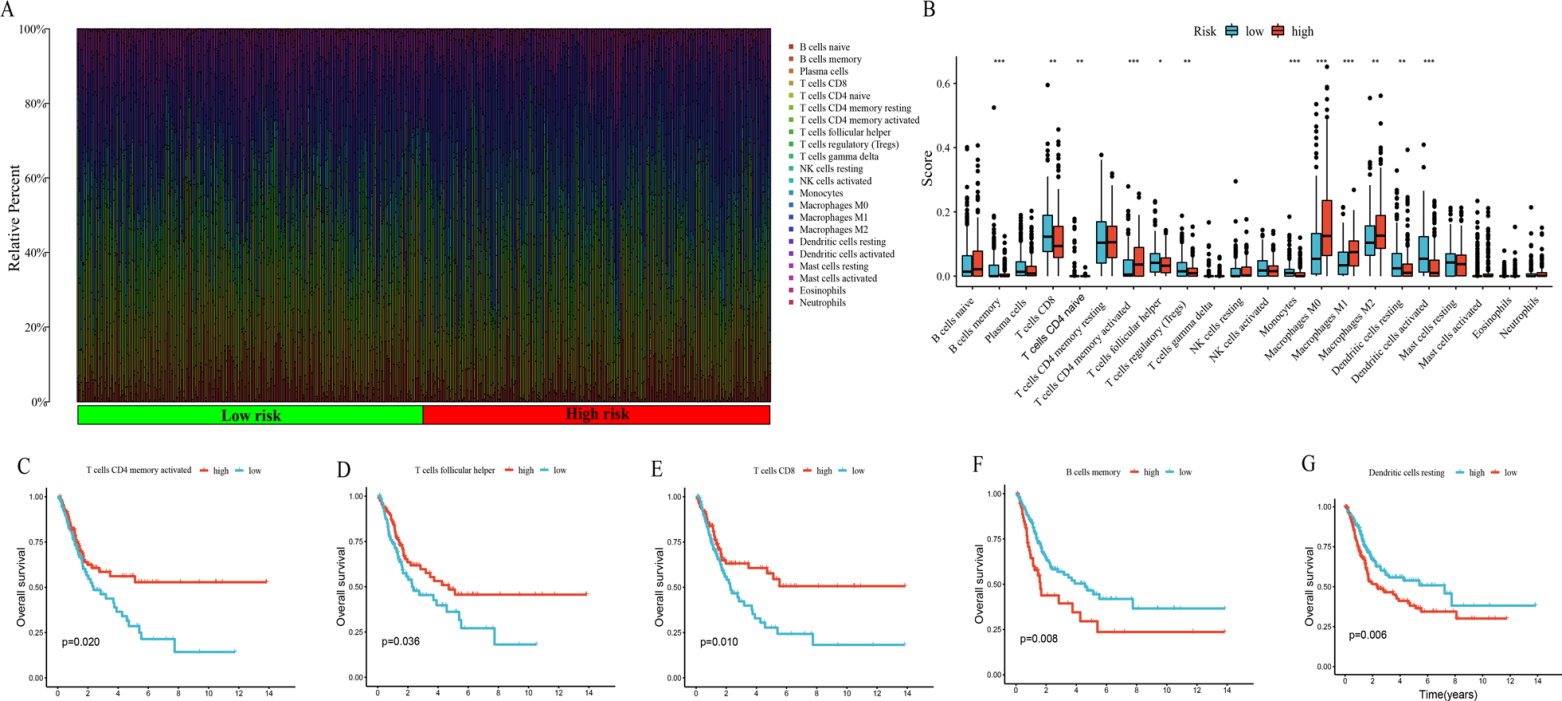
Correlation between the fraction of 22 TIICs and the prognostic model. **A** Proportion of 22 TIICs in BLCA. **B** Differential analysis of 22 TIIC fractions between risk score-defined groups. **C**–**G** Association between the infiltration level of TIICs [activated CD4 memory T cells (**C**), follicular helper T cells (**D**), CD8T cells (**E**), memory B cells (**F**), resting dendritic cells (**G**)] and OS of BLCA patients. *p < 0.05; **p < 0.01; ***p < 0.001, ns = no significance

Next, the CIBERSORT algorithm was applied to calculate the fraction of 22 TIICs in each TCGA-BLCA sample. A grouping histogram depicting the distribution of TIICs in BLCA (Fig. 8A). Then, we fund that the fractions of activated CD4 memory T cell, M0, M1 and M2 Macrophages were significantly higher in the high-risk group (Fig. 8B), while memory B cells, CD8 T cells, naïve CD4 T cells, follicular helper T cells (Tfh), regulatory T cells (Tregs), monocytes, resting and activated dendritic cells (DCs) were significantly higher in the low-risk group (Fig. 8B). Higher proportions of memory B cells and lower fractions of activated CD4 memory T cell, Tfh, CD8 T cells and resting DCs among these differently distributed TIICs were substantially linked with poor OS in BLCA patients (Fig. 8C–G). Hence, we believe necroptosis might regulate these TIICs to affect the prognosis of BLCA patients.

### Correlation between the prognostic model and somatic mutation and drug sensitivity

**Fig. 9:**
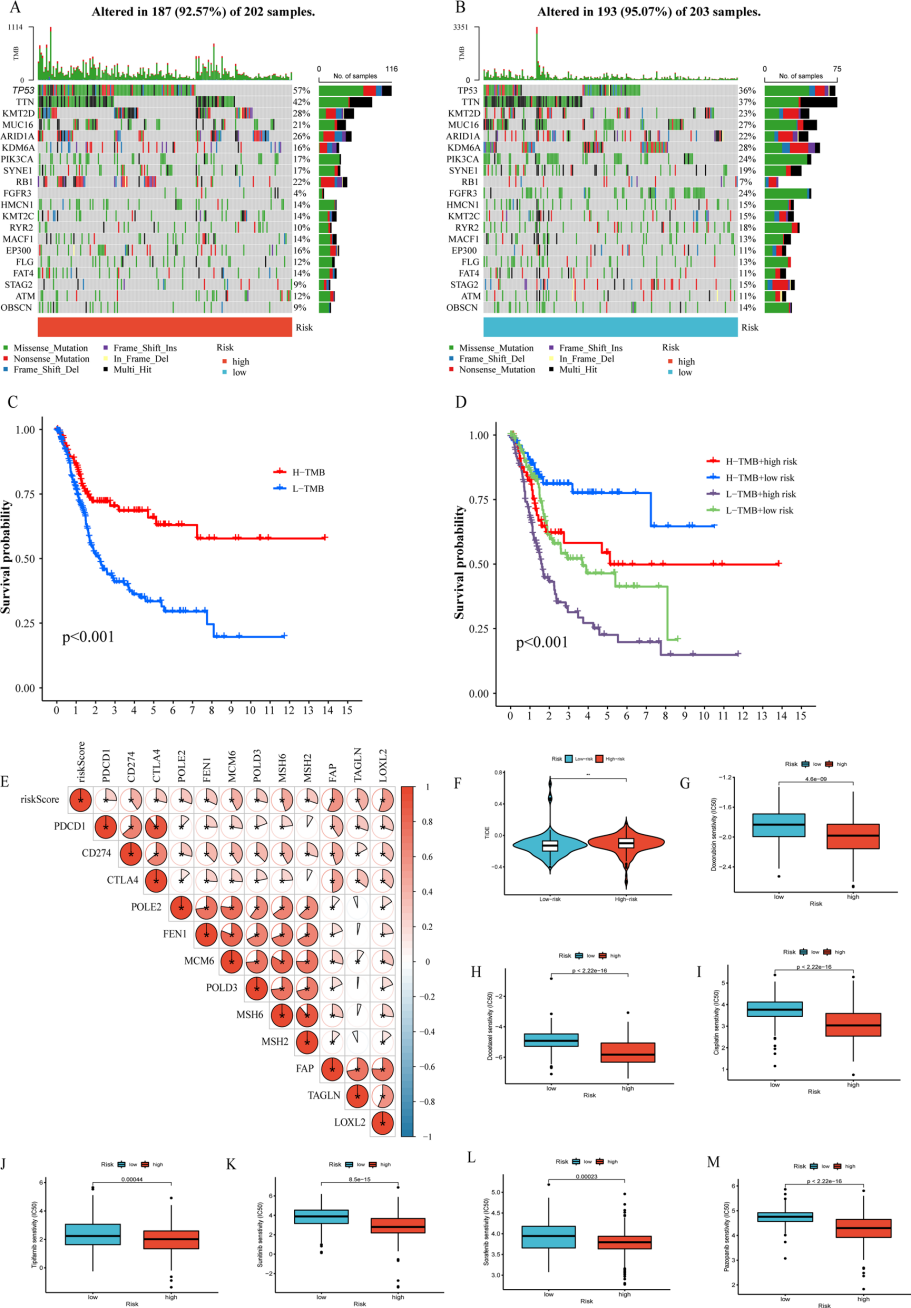
Correlation between the prognostic model and somatic mutation and drug sensitivity. **A**, **B** Waterfall plots of 20 genes with the highest mutation rate in the high-risk group (**A**) and low-risk group (**B**). Kaplan–Meier analysis of TMB in BLCA patients based on TMB defined groups (**C**) and risk score with TMB-defined groups (**D**). **E** Correlation between expression of ICGs and risk score. **F** TIDE score. Correlation between the Prognostic model and IC_50_ values of chemotherapy and immunotherapy drugs, including doxorubicin (**G**), docetaxel (**H**), cisplatin (**I**), tipifarnib (**J**), sunitinib (**K**), sorafenib (**L**) and pazopanib (**M**)

There was a lot of evidence that tumorigenesis was associated with accumulation of gene mutations. Figure 9A, B revealed simple nucleotide variation of risk score groups in BLCA cases, suggesting that the 20 genes with the highest mutation rate in BLCA were TP53, TTN, KMT2D, MUC16, ARID1A, KDM6A, PIK3CA, SYNE1, RB1, FGFR3, HMCN1, KMT2C, RYR2, MACF1, EP300, FLG, FAT4, STAG2, ATM and OBSCN. Then, to split patients into low- and high-TMB groups, we determine the best TMB cutoff value. BLCA patients with lower TMB were associate with poorer OS (Fig. 9C). In addition, BLCA patients with lower TMB and higher risk scores have poorer OS survival probability, whereas BLCA patients with higher TMB and lower risk scores have greater OS (Fig. 9D).

In addition, we investigated potential correlations between ICGs expression and risk score. Figure 9E showed that risk score was significantly positively associated with the expression of PDCD1, CTLA4, POLE2, FEN1, MCM6, POLD3, CD274, MSH6, FAP and LOXL2. And the TIDE score was significantly lower in low-risk group (Fig. 9F). As a result of the abovementioned, immunotherapy was more likely to benefit BLCA patients in the high-risk group.

Next, we calculated IC50 values to predict risk score for chemotherapy and immunotherapy drugs. The IC50 value of doxorubicin, docetaxel, cisplatin, tipifarnib, sunitinib, sorafenib and pazopanib was notably lower in the high-risk group, suggesting that BLCA patients with high-risk score were more benefit from these drugs (Fig. 9G–M).

### Functions of the identified biomarker in bladder cancer progression

**Fig. 10:**
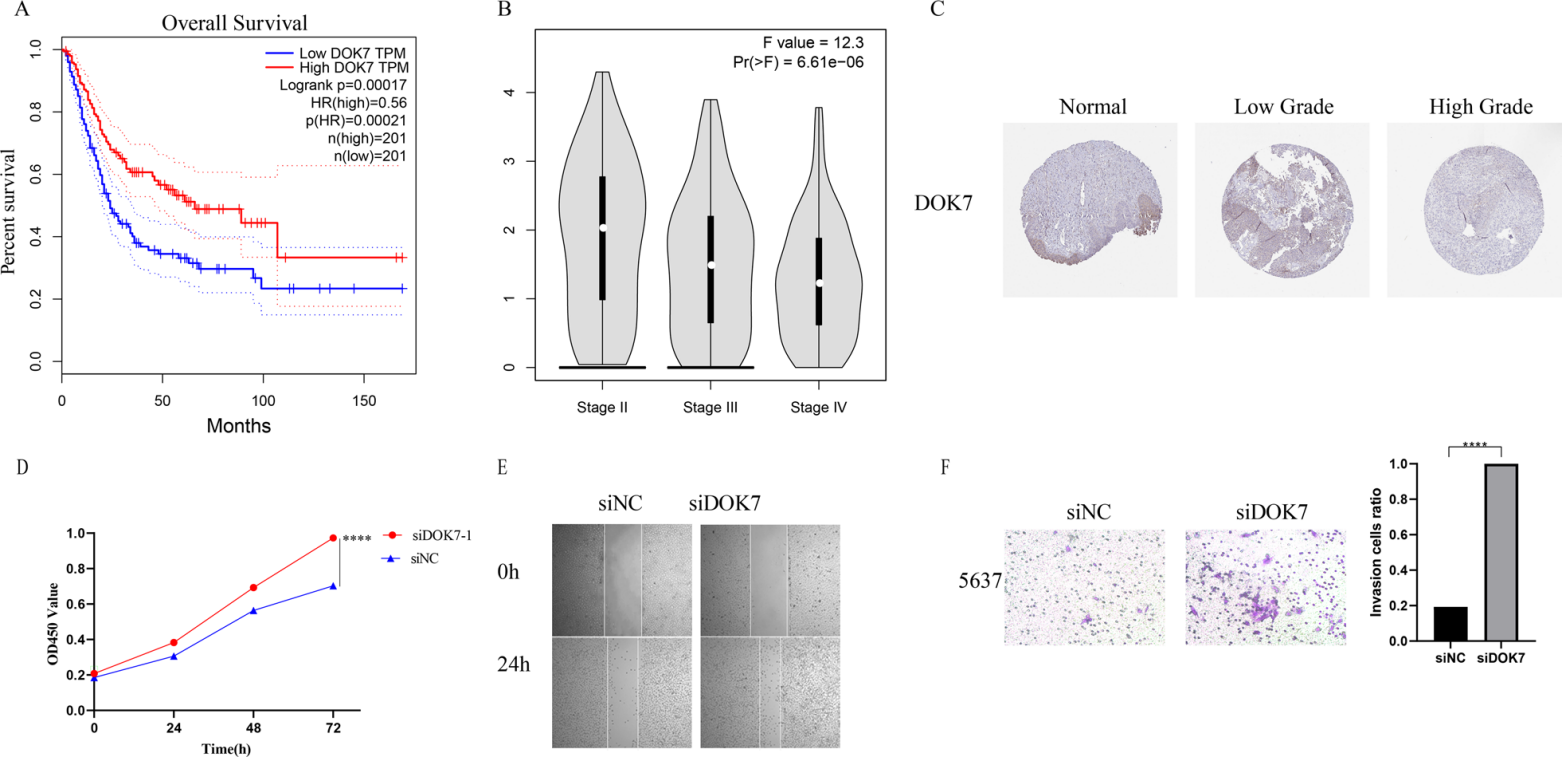
Functions of the identified biomarker in bladder cancer progression. **A** Relationship between the expression level of DOK7 and OS in TCGA. **B** Relationship between the expression level of DOK7 and cancer stage in TCGA. **C** Immunohistochemical (IHC) analysis of DOK7 in normal bladder tissues and tumor tissues with different grades of malignancy. **D**–**F** CCK-8, wound healing and transwell migration assay for analyzing the effect of DOK7 knockdown on cell proliferation and migration

Five genes in our prognostic model, ANXA1, MAP1B and SPOCD1 have been reported in bladder cancer, however DOK7 and FKBP10 has not been studied in bladder cancer [30–33]. There was reported that DOK7 could inhibit breast cancer cell invasion and migration ability via PI3K/PTEN/AKT pathway [34], and in our above results, the DEGs were related to PI3K/AKT signaling pathway. So, we chose DOK7 to further verify our prognostic model. According to the data of TCGA, patients who have higher expression of DOK7 have a better OS, and with the staging increase the expression of DOK7 has decreased (Fig. 10A, B). In addition, we investigated the protein expression of DOK7 in high grade tumor, low grade tumor and normal bladder tissues respectively by using the HPA dataset, and immunohistochemical staining indicated the positive staining intensity of DOK7 in normal bladder tissues as notably stronger than BLCA tissues. Moreover, tumor samples with a low pathological grade revealed stronger expression than tissues with a high grade by HPA (Fig. 10C).

Last, functional studies were performed by using small interfering RNAs (siRNAs) to knockdown of DOK7, and the results indicated that the knockdown of DOK7 could promote the cell proliferation and migration ability (Fig. 10D–F). All of the results were consistent with our prognostic model.

## Discussion

Despite significant advancements in diagnosis and therapy, bladder cancer remains a serious clinical problem due to high rates of recurrence and metastasis [4]. As a result, optimizing therapy regimens to reduce BLCA patient mortality is critical.

There are several prognostic models about bladder cancer have been explored, and they classified bladder cancer with pyroptosis-related genes, ferroptosis-related genes, and neutrophil-related genes or m6A-immune-related lncRNA [35–38]. But our prognostic model is based on necroptosis-related genes in bladder cancer, and to predict the prognosis and treatment of bladder cancer.

In our study, by consensus clustering, we uncovered two necroptosis-related patterns using BLCA samples from the TCGA-BLCA. Cluster B has a much lower OS survival rate and a significantly higher percentage of patients in advanced clinicopathological stages. In addition, several immune checkpoints were discovered to be substantially expressed in Cluster B (PDCD1, CD274, IDO1, PDCD1LG2, LAG3, TIGIT and CTLA4). Furthermore, TME immune cell infiltration and biological pathway enrichment differ between these two necroptosis-related patterns. Cluster B was distinguished by significant levels of TME immune cell immersion and adaptive immunity activation in this pattern. Based on above results, we hypothesized that immune checkpoints genes expressed high-level could strengthen T cell activation and activate the immune pathway to weak the effects of tumor suppression and elimination [39], and we believed that necroptosis may has a critical role in BLCA’s immune landscape regulation and may be a prognosis predictor.

Next, we established a prognostic model including five genes (ANXA1, DOK7, FKBP10, MAP1B and SPOCD1) to obtain a metrics that can forecast the clinical survival rate of BLCA patients accurately and effectively. A series of analyses indicated that BLCA patients in high-risk groups have poorer OS and prognosis event in the TCGA-BLCA cohort. And it was consistently verified in GSE13507 cohort. The risk score was subsequently validated using univariate and multivariate Cox regression analysis, indicating that it may be utilized as a reliable independent prognostic indicator for BLCA patients. ANXA1 is a member of annexins superfamily, and plays a role in inflammation regulation and can influence T cell proliferation [40, 41]. ANXA1 also could promotes the progression and drug resistance in bladder cancer [30, 42]. DOK7 is a docking protein correlate with tumor recurrent and an indicator of cancer risk [43–45]. Overexpression of FKBP10 is find to boost cancer progression by restrict antitumor immunity or activate tumor-related signaling pathways [46, 47]. MAP1B, one of microtubule-associated proteins (MAPs), is reported as the most significant upregulated gene in urothelial carcinoma progression [31]. And phosphorylation of MAP1B associate with drug sensitive in human glioblastoma [48]. SPOCD1 could inhibits cell apoptosis through PI3K/AKT pathway and accelerates ovarian cancer progression [49]. However, DOK7 and FKBP10 have not been reported as predictor of BLCA patients. Because of DOK7 could inhibit breast cancer cell invasion and migration ability via PI3K/PTEN/AKT pathway, and our result showed a similar pathway. Based on that found, we thought that DOK7 may also could prompt or inhibit bladder cancer. According to the data of TCGA and HPA, the higher expression of DOK7 indicated a bad prognosis, and the results were consistent with our prognostic model. To further verify the functions of DOK7 in bladder cancer, we used siRNA to knockdown the expression of DOK7. Consistently, with the knockdown of DOK7, bladder cancer cell proliferation and migration abilities were promoted.

In consideration of the powerful inflammatory, we explored the correlation between TME and the risk score. Our result showed the high-risk group has a considerably higher StromalScore, ImmuneScore and ESTIMATEScore, indicating that their tumor purity was lower and may associated with poor prognosis [50]. In addition, the fractions of activated CD4 memory T cell, M0, M1 and M2 Macrophages were significantly higher in the high-risk group, while memory B cells, CD8 T cells, naïve CD4 T cells, Tfh, Tregs, monocytes, resting and activated dendritic cells were significantly higher in the low-risk group. Several research reported that the infiltration of memory B cells, CD8 T cells DCs, and Tfh cells in tumor may associate with better prognosis [51–54], while M2 macrophage infiltration correlates with chemotherapy resistance and is associated with a poor prognosis in most cancers [55, 56]. Besides, monocytes also could activate antigen-presenting cells to play a role of antitumor effectors [57]. Other study report Tregs could promote tumor progression by form an immunosuppressive microenvironment [58]. But in our study memory B cells with high components of the TME and CD4 memory T cell with low components of the TME are mean poor prognosis. The conflict requires further investigation in order to be fully understood.

Gene mutations play a critical part in the development of BLCA, and somatic mutations are considered the primary drivers of antitumor adaptive immune response [59]. Our study demonstrated BLCA patients with higher TMB have a better OS survival probability. We also combine risk score and TMB to predict the OS of BLCA patients, and the result show that BLCA patients with low-risk score and high TMB have the greatest OS survival probability. Immune checkpoint inhibitors (ICIs) efficacy are reported have a correlation with TMB, and cancer patients with high TMB seem like to have better reaction to ICIs [60, 61]. Next, we assessed the prognostic model’s ability to predict chemotherapy and immunotherapy drug benefit in BLCA patients. The result showed that compared with the low-risk group, doxorubicin, docetaxel, cisplatin, tipifarnib, sunitinib, sorafenib and pazopanib were significantly benefit in the high-risk group. When taken as a whole, the prognostic model may provide better therapy strategies for BLCA patients.

There are still some limits to our research. First, the samples are constrained because of sample size and sample origin from public datasets. Second, the prognostic model needs clinical studies to confirm its accuracy and stability. Moreover, RNA expression is not completely present protein level. As a result, more research is required to overcome these limitations.

## Conclusion

In summary, our research identifies a novel prognostic model for predicting prognosis of BLCA patients. Based on our prognostic model, we believe that we could make an accurate judgment based on the different conditions of bladder cancer patients and could provide references for individualized treatment of chemotherapy and immunotherapy drugs.

## Data Availability

The datasets supporting the conclusions of this article are available in The Cancer Genome Atlas database (https://portal.gdc.cancer. gov/), the GEO database (https://www.ncbi.nlm.nih.gov/geo/) and HPA (http://www.proteinatlas.org).
